# CRISPR/Cas9 System for Efficient Genome Editing and Targeting in the Mouse NIH/3T3 Cells

**Published:** 2019

**Authors:** Maryam Mehravar, Abolfazl Shirazi, Mohammad Mehdi Mehrazar, Mahboobeh Nazari, Mehdi Banan

**Affiliations:** 1. Reproductive Biotechnology Research Center, Avicenna Research Institute, ACECR, Tehran, Iran; 2. Monoclonal Antibody Research Center, Avicenna Research Institute, ACECR, Tehran, Iran; 3. Genetics Research Center, University of Social Welfare and Rehabilitation Sciences, Tehran, Iran; 4. Research Institute of Animal Embryo Technology, Shahrekord University, Shahrekord, Iran

**Keywords:** Cell line, Deletion, Gene editing, Mice

## Abstract

**Background:**

The Clustered, Regularly Interspaced, Short Palindromic Repeats (CRIS-PR) and CRISPR-associated protein (Cas) system has been used as a powerful tool for genome engineering. In this study, the application of this system is reported for targeting *Rag* genes to produce mutant mouse NIH/3T3 cell line. The *Rag*1 and *Rag*2 genes are essential for generation of mature B and T lymphocytes. Disruption of *Rag* genes causes disease like Severe Combined Immunodeficiency syndrome (SCID). Here, the efficiency and specificity of CRISPR system were tested with highly active sgRNAs to generate novel mutations in the NIH/3T3 mouse cell line.

**Methods:**

Four single guide RNAs were designed to target sequences in the coding region of the *Rag*1 and *Rag*2 genes. Four sgRNA-CAS9 plasmids were tested to target *Rag1* and *Rag2*.

**Results:**

Based on T7 endonuclease assay and sequencing analysis, the expression of sgRNAs targeting two sites in *Rag*1 resulted in deletion of the intervening DNA fragment. The expression of sgRNAs with Cas9 targeting two sites in *Rag*2 gene resulted in indel mutations at both sites. In this report, fragment deletion in *Rag*1 gene was detected in about 50% of transfected cells.

**Conclusion:**

Therefore, CRISPR/Cas9 system can be highly efficient and specific when gRNAs are designed rationally and provides a powerful approach for genetic engineering of cells and model animals.

## Introduction

Recently, the Clustered Regularly Interspaced Short Palindromic Repeats (CRISPR) system associated to the Cas9 endonuclease (CRISPR/Cas9) has been developed as a specific and effective tool for genome engineering. Compared to other genome editing technologies based on endonucleases such as Zinc Finger Nuclease (ZFN) and Transcription Activator-Like Effector Nuclease (TALEN) that are rather complex to design and need to be assembled for each target sequence, the CRISPR/Cas9 is inexpensive and easy to carry out 1–5.

This system is based on the base-pairing of the genomic sequence, adjacent to an obligate protospacer adjacent motif (PAM) NGG, with a short complimentary RNA sequence which is then cleaved by the Cas9 protein in a sequence-specific manner 5–7. Double Strand Breaks (DSBs) induced by this system can be repaired in one of the two ways: Non-Homologous End Joining (NHEJ) or Homology-Directed Repair (HDR), resulting in insertions or deletions in desired genomic regions. Deletions may result from introducing two sg-RNAs along with Cas9. The sgRNAs induce two DSBs and deletion of the intervening fragment may occur. There are several advantages of genomic deletions compared to single-site small indels including:

1. easy identification by conventional PCR, while identification of small indels or point mutations may require time consuming techniques such as Restriction Fragment Length Polymorphism (RFLP), immunoblotting, T7 Endonuclease (T7EN1) cleavage assay and
2. predictability loss of gene function 8.

The CRISPR-Cas system has been used for genome editing in several organisms 4,5,9–13 and mammalian cell lines 14–17. Although genome editing by CRISPR-Cas system has been demonstrated to be non-specific in some cases 18, it can be improved in specificity if gRNAs are designed correctly.

Currently, a variety of animal models with natural immune system defects can be applied for the immunology and genetic studies 19. The *Rag*1 and *Rag*2 genes play an important role in the rearrangement and recombination of immunoglobulin and T-cell receptor genes during the process of V(D)J recombination. In mouse models with *RAG*-1 and *RAG*-2 deficiency, deletion of the *Rag*-1/*Rag*-2 genes cause the arrest of rearrangement of B-cell receptors (Immunoglobulin production) and T-cell receptors and lack of the T and B cell differentiation.

In recent years, programmable site-specific nucleases have been used to generate recombination-activating genes (*RAG*1/*RAG*2) immunodeficient mouse models 20–22. As mentioned above, the *RAG* proteins are essential for the V(D)J recombination process, which generates diversity of immune systems. *RAG*-1/*RAG*-2 deficient mice do not produce mature B and T lymphocytes. The loss of *RAG* function leads to a Severe Combined Immunodeficiency (SCID) phenotype 23.

In this study, knock-out mouse cell line models of *RAG*1/*RAG*2 were created. The following procedures were performed; identification of appropriate target sites on *Rag* genes to design sgRNAs based on rules handling on-target efficacy, construction of CRISPR/CAS9 plasmids by cloning strategy, co-transfection of CRISPR/CAS9 plasmids and puromycin resistance vector into mouse model NIH/3T3cell lines and finally analysis of mutagenesis induced by CRISPR system in transfected cell line.

## Materials and Methods

### Target site selection

Target sequences G/A-(N19)-NGG were selected for U6 plasmid-base transcription. The target sequence can be present on either DNA strand. To choose the most specific target for sgRNA, the potential for off-target effects should be minimized. For this purpose, there are several computational tools allowing the design of sg-RNA target sequences with minimum potential for off-targets 24–26.

In our study, assessment of off-target searching was performed using the online CRISPR RGEN Tools program (https://www.rgenome.net). Therefore, target selection was done based on following criteria; 5′-G-N_19_-NGG-3′ matching and selection of target sequences in coding region nearby gene promoter. Finally, four target sequences in coding regions of *Rag*1 and *Rag*2 genes were selected where two sequences targeted the *Rag*1 gene and two other sequences targeted the *Rag*2 gene, respectively.

### sgRNA design

Finally, based on the mentioned rules for target selection, sgRNA sequences were ordered in the form of phosphorylated forward and reverse oligonucleotides flanked by desired restriction sites. These sgRNA oligonucleotides can be annealed to generate linkers compatible with restriction sites of digested sgRNA/Cas9 expressing vector.

### Construction of sgRNA and Cas9 expressing vector

In our experiment, two kinds of CRISPR expressing vectors were used: pX330 vector (Addgene#42230) and pLenti-Cas-Guide vector (Origene, GE100010) to express codon optimized spCas9 under CMV (in pX-330) or CBh (in pLenti-Cas-Guide) promoter and sg-RNA under U6 promoter. Antibiotic resistance genes used as the selection markers were ampicillin and chloramphenicol in pX330 and pLenti-Cas-Guide vectors, respectively.

In order to target four sequences in coding regions of *Rag*1 and *Rag*2 genes, desired sgRNA/Cas9 vectors were constructed. To clone a sgRNA into pX330 expression vector, the vector was digested with *Bbs*I (Thermo scientific, ER1011). Similarly, the pLenti-Cas-Guide vector was digested with *Bam*HI (Thermo scientific, ER0051) and *Bsm*bI (Thermo scientific, ER-0451) restriction enzymes, then both vectors were treated with Alkaline Phosphatase (Thermo scientific, EL0011). Complementary oligos of *RAG*1-F1 *RAG*1-R1, *RAG*1-F2 *RAG*1-R2, *RAG*2-F1 *RAG*2-R1 and *RAG*2-F2 *RAG*2-R2 (Table 1) for each target sequence were heated at 95*°C* for 5 *min*, and annealed by decreasing the temperature from 0.5*°C/s* to 22*°C* using a thermocycler (Eppendorf, USA). Then, the short double strand DNA fragments (*RAG1*-F1R1, *RAG2*-F1R1 and *RAG2*-F2R2) were ligated into linearized *Bbs*I site of pX330 and *RAG*1-F2R2 was ligated into linearized *Bam*HI and *Bsmb*I sites of pLenti-Cas-Guide vector. Ligation products were transformed into *Escherichia coli (E. coli)* DH5α competent cells to get clones. *E. coli* were grown in LB (Luria-Bertani) medium containing desired antibiotics as selective markers.

**Table 1.** Oligonucleotides used to generate the sgRNA plasmids for four different mouse ORFs: *RAG* 1-F1R1, *RAG* 1-F2R2, *RAG* 2-F1R1, *RAG* 2-F2R2; underlined letters show the flanked desired restriction sites; red letters show the restriction site in *RAG* 1-F2R2

**sgRNA names**	**Sequence 5′ to 3′**	**Restriction site**
***RAG*1-F1**	CACCGTGCGACGGTCCCGTCTCGCG	
***RAG*1-R1**	AAACCGCGAGACGGGACCGTCGCAC	
***RAG*1-F2**	GATCGCATGGCAGAATTCCGTCGGGG	*EcoRI*
***RAG*1-R2**	AAAACCCCGACGGAATTCTGCCATGC	*EcoRI*
***RAG*2-F1**	CACCGGAATGGCCGTATCTGGGTTC	
***RAG*2-R1**	AAACGAACCCAGATACGGCCATTCC	
***RAG*2-F2**	CACCGGTATAGTCGAGGGAAAAGCA	
***RAG*2-R2**	AAACTGCTTTTCCCTCGACTATACC	

### Restriction enzyme digestion and sequencing

In order to confirm ligation and correct direction of inserts, digestion with restriction enzymes and sequencing were performed. For the restriction enzyme digestion, the cloned pX330 vector (In which *Bbs*I restriction site has been removed after cloning) was digested with *Bbs*I restriction enzyme and the cloned pLenti-Cas-Guide vector (Since *RAG*1-F2R2 sgRNA contains *Eco*RI restriction site, so the new construct presents with an additional *EcoRI* restriction site) was digested with *Eco*RI. For the sequencing, desired primers for regions surrounding the target sites in *Rag*1 and *Rag*2 genes were designed (Table 2). The sequencing results were analyzed by sequence alignment program clustalW2 to confirm correct direction of inserts.

**Table 2.** Primers used to amplify fragments of *RAG1* and *RAG2* genes

**Primer names**	**Sequence 5′ to 3′**
***RAG*1-primer F**	GAA GAA GCA CAG AAG GAG AAG
***RAG*1-primer R**	ATC GGC AAG AGG GAC AAT AGC
***RAG*2-primer F**	ATTCCTCCTGGCAAGACT
***RAG*2-primer R**	GCATAGACTCTGACAAGCA

### Transfection of Cas9-sgRNA vector into NIH/3T3 cell line

Among reagents for lipofection, lipofectamine 2000 has the highest efficiency for NIH/3T3 transfection^27^. In our experiments, pLKO.1-puro vector (Addgene #10878) containing puromycin resistance gene was used for the selection of cells that have been transfected by sgRNA-Cas9 vector because pX330 and pLenti plasmids do not contain any markers for selection. Therefore, NIH/3T3 cell lines were transiently co-transfected with pLKO.1-puro vector and Cas9-sgRNA vector using lipofectamine 2000.

### Cell culture and Cas9-sgRNA vector transfection

NIH/3T3 cell lines were grown in DMEM media supplemented with 10% FBS. Cells were co-transfected with sgRNA-Cas9 expressing vector and pLK-O.1 vector using lipofectamine 2000 (Invitrogen, 11668**)** according to manufacturer's protocol. For this purpose, one day prior to transfection, the cells were seeded in a 6-well plate at a cell density of 2×10^5^ cells per well until they reached ∼50% confluence. For plasmid DNA transfection, 2.5 *μg* DNA (1:9 puromycin: sgRNA ratio) was added to 150 *μl* of Opti-MEM medium (Invitrogen, 31958), followed by addition of 150 *μl* of Opti-MEM containing 5 *μl* of Lipofectamine 2000. The sample was mixed by gently flicking the tubes a few times and then the mixture was incubated at room temperature for 20 *min*. The entire solution was added to the cells in a 6-well plate and mixed by gently swirling the plate. The plate was incubated at 37*°C* for 48 *hr* in a 5% CO_2_ incubator. Medium waschanged 48 *hr* after transfection with a fresh medium containing 7.5 *μg* puromycin for each well. Puromycin selection was done over 3–5 days. After selection, transfected cells were assayed. To monitor transfection efficiency, transfection control wells (GFP plasmid) were used.

### T7E1 mismatch-detectingassay

Genomic DNA from transfected and control NIH/3T3 cells was isolated by DNA extraction kit (Qiagen). Fragments containing *Rag*1 and *Rag*2 genes were amplified from extracted DNA using *Rag*1 and *Rag*2 primers that had been designed in the vicinity of the target sites in *Rag*1 and *Rag*2 genes, respectively (Table 2). Purified PCR products (100 *ng*) were denatured and re-annealed under the following thermocycler conditions: 95*°C* for 5 *min*, 95 to 85*°C* at −2*°C/s*, 85 to 25*°C* at −0.1*°C/s*, and held at 4*°C*. Then, 2 *μl* of NEB buffer 2 (New England Biolabs, Ipswich, MA, USA), 1 *μl* (5 *U*) of T7 endonuclease I (New England Biolabs, M-0302S), and H_2_O were added to total volume of 20 *μl* and the mixture was incubated at 37*°C* for 30 *min*. Finally, treated PCR products were analyzed by 2% agarose gel to visualize the cleavage bands. The non-transfected control sample was also treated by T7E1 and used as a negative control, since sometimes minor background bands may be present even in the negative control sample due to the unspecific cleavage of T7EI nuclease 28,29.

### Sequencing analysis

The PCR products were sequenced by Sanger sequencing with desired *Rag*1 and *Rag*2 primers and mutations were confirmed by sequencing (Macrogen, Korea).

## Results

### Target selection, design and characterization of Cas9 and sgRNAs expressing vector

To design effective sgRNAs for editing the mouse *Rag*1/*Rag*2 genes, the sequence within coding regions of *Rag*1/*Rag*2 genes was analyzed through the online CRISPR RGEN Tools program (https://www.rgenome.net).

**Figure 1.**
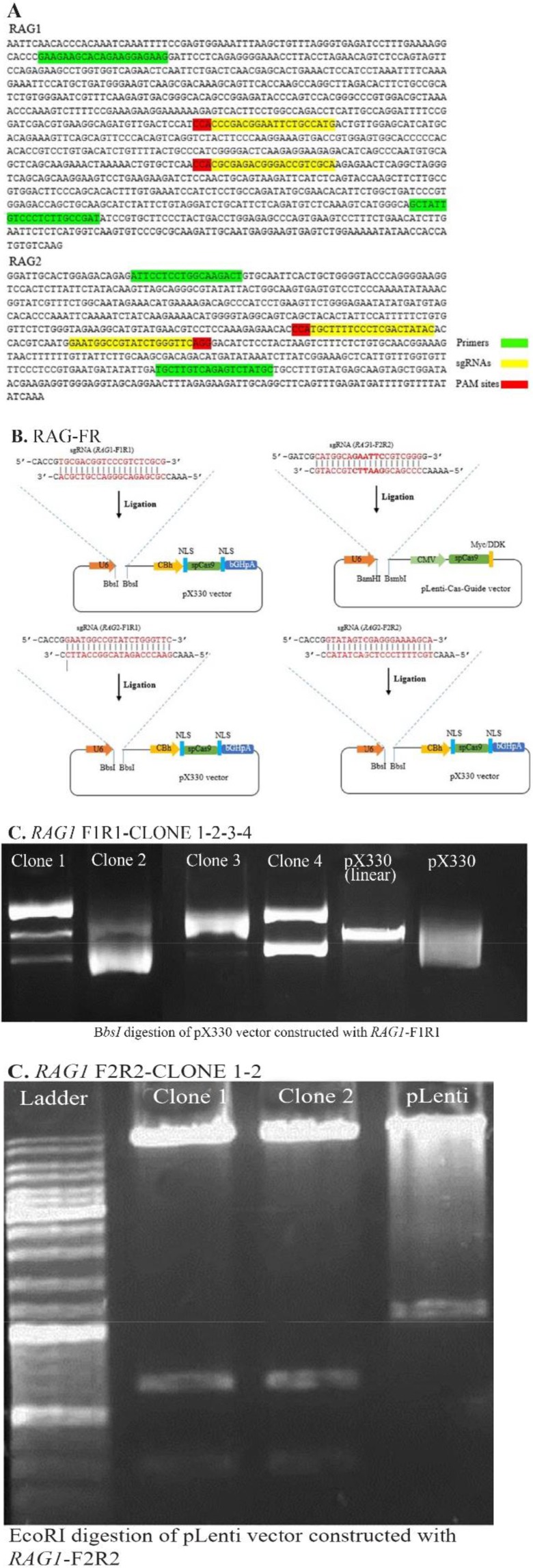

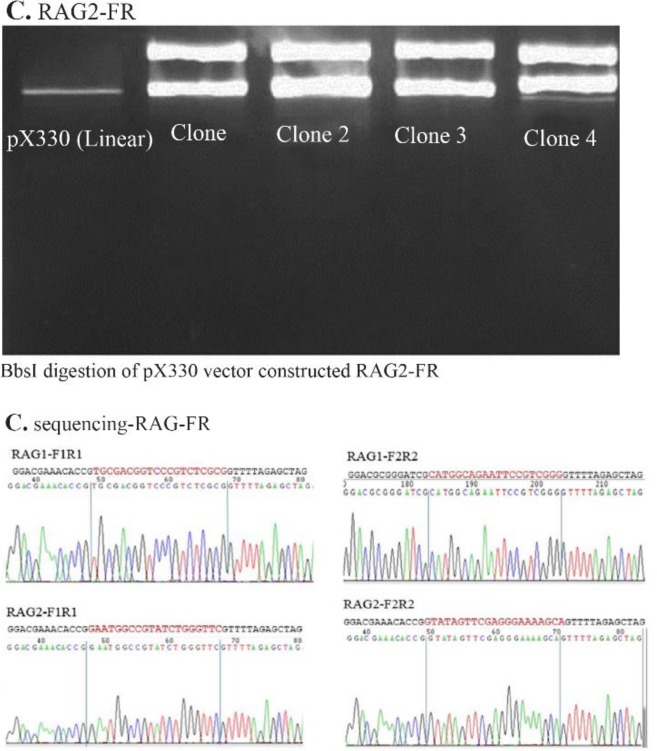
Target selection and construction of Cas9 and sgRNAs expressing vector. A) Sequences corresponding to the *Rag*1 and *Rag*2 fragments used for the selection of *RAG* primers and targeted sites. Primer sequences and target sites are respectively highlighted in green and yellow. B) Construction of pX330 and pLenti-Cas-Guide vectors with *RAG*1 and *RAG*2 sgRNAs. *RAG1*-F1R1, *RAG2*-F1R1 and *RAG2*-F2R2 were inserted into the *Bbs*I site of pX330 and *RAG1*-F2R2 was inserted into the *Bam*HI and *Bsmb*I sites of pLenti-Cas-Guide vector by cloning strategy. Cloned sgRNA will be driven by U6 promoter, and Cas9 expression will be driven by CBh (in pX330) and CMV (In pLenti-Cas-Guide) promoters. Ampicillin (In pX330) and chloramphenicol (In pLenti-Cas-Guide) resistance gene can be used to enrich transfected cells. C) Above: Gel-electrogram images of restriction enzyme digestion of vectors. *Bbs*I digestion of pX330 plasmid and *EcoR*I digestion of pLenti-Cas-Guide plasmid showing correct vector assembly in bacterial clones. Constructs of clones with correct digestion were sequenced. Below: Sequencing results. Example of chromatogram showing correct cloning of oligos into vectors.

We targeted two regions in each *Rag*1/*Rag*2 gene in which two sgRNAs separated by 213 *bp* for *Rag*1 and 2 sgRNAs separated by 12 *bp* for *Rag*2 (Figure 1A). Potential target sites were selected based on predictive rules to avoid off-179 targets (see Materials and Methods). We targeted two regions in each *Rag*1/*Rag*2 gene in which two 180 sgRNAs were designed.

We assembled sgRNAs in separate expression vectors using cloning steps as described in materials and methods. Only plasmid DNA was usedto transfect, since RNA preparation, handling and storage take laborious steps. Four sgRNAs were constructed into vectors with sgRNA scaffolds driven by a U6 promoter and Cas9 under CBh and CMV promoter in pX330 and pLenti-Cas-Guide, respectively (Figure 1B). To verify ligation, sequencing and diagnostic restriction digestion were performed (Figure 1C). pX330 has been designed so that when the plasmid is cloned with *ad hoc* sgRNA, *Bbs*I restriction site is lost. Conversely, cloning the guide sequence into the pLenti vector generates an additional *Eco*RI restriction site. The results of sequencing and diagnostic digestion demonstrated that sgRNAs have been successfully constructed in CRIS-PR/Cas9 vectors. For constructing *RAG*1-F1R1/pX330, among several clones only two clones (clone 2 and 4), for constructing *RAG*1-F2R2/pLenti, clone 1 and 2, for constructing *RAG*2-F1R1/pX330, clone 4 and 5 had the correct ligation based on the results of restriction enzyme digestion and sequencing. For constructing *RAG-*1-F2R2/pX330, clone 1 and 2 had the correct ligation based on the results of restriction enzyme digestion but only clone 2 had the correct insert direction based on the results of sequencing.

### sgRNA-Cas9 guided genome editing in Rag genes

**Figure 2.**
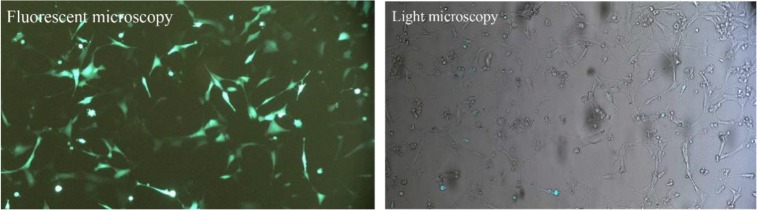
Examination of transfection efficiency by fluorescent microscope. Left: Fluorescent microscopy image of NIH/3T3 cells transfected by GFP. Right: The image of the same GFP-transfected NIH/3T3 cells by light microscope.

**Figure 3.**
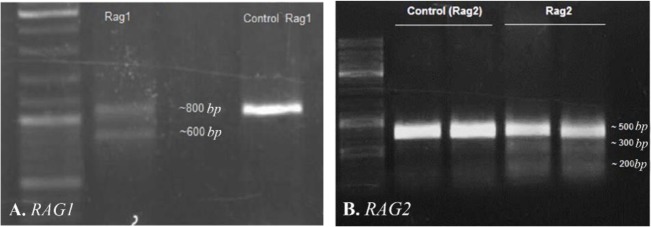
Identification of CRISPR-mediated cleavage activity. A) Gel-electrogram image of *RAG1* fragments after CRISPR-mediated cleavage activity. PCR products of Rag1 were amplified and directly analyzed by 2% agarose gel. The presence of ∼600 *bp* fragment showed that 213 *bp* fragment has been deleted from 800 *bpRAG*1 fragment. B) Gel-electrogram image of *RAG*2 fragments. After CRISPR-mediated cleavage activity, PCR products of *RAG*2 were amplified and subjected to T7EN1 cleavage assay. Cleavage bands were marked with an asterisk “*”.

*Rag*1 and *Rag*2 sgRNAs targeted two sites simultaneously in each gene. To analyze genome editing by sgRNA-Cas9, genomic DNA was isolated from cells harvested 3–5 days after transient transfection. The rate of transfection was visualized about 50% based on EGFP fluorescence using fluorescent microscopy (Figure 2). Extracted DNA was analyzed for the presence of site-specific gene modification by PCR amplification of regions surrounding the target sites as well as T7EN1 cleavage assay. Fragments resulted from *Rag*1 PCR amplification showed that before T7EN1 cleavage assay, there were two bands of *Rag*1 fragment on agarose gel in transfected lane compared with wild type lane. This smaller amplicon was the result of the expected deletion that occurred in the *Rag*1 gene in a large number of cells (Figure 3A). Since gel electrophoresis after cleavage activity showed the occurrence of the fragment deletion in *Rag*1 gene, there was no need to subject the PCR products of *Rag*1 to T7EN1 cleavage assay. Deleted fragment ∼213 *bp* in size could rapidly be detected by PCR without requirement for T7EN1 cleavage assay since deletion has occurred in many cells. Evidence of nuclease cleavage of *Rag2* gene could not be detected by PCR amplification and only was visible after T7EN1 cleavage assay (Figure 3B).

### Sequencing of PCR product

**Figure 4.**
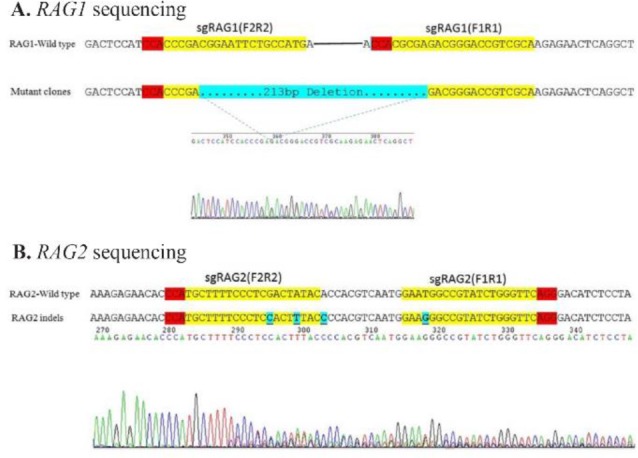
Analysis of CRISPR-mediated mutations by Sanger sequencing. A) Sequencing of PCR products of RAG1 sgRNA target site shows the expected deletion. B) Sequencing of PCR products of RAG2 sgRNA target site shows the expected mutations. Target site is indicated in yellow, PAM sequence in red and cleavage sites are in blue.

To confirm the results of cleavage assay, Sanger sequencing was used and showed the indels were detected at target sites (Figure 4).

## Discussion

In the present study, it was shown that genome editing by CRISPR/Cas9 system is efficient with highly active sgRNAs to generate novel mutations of *Rag*1/*Rag*2 genes in the NIH/3T3 mouse cell line.

Amazing result for *Rag*1 gene showed that before T7EN1 cleavage assay, only by a simple PCR reaction an obvious deletion was detected in *Rag*1 gene in a large number of cells. These results of mutation detection in *Rag*1/*Rag*2 genes demonstrate that CRISPR/Cas9 system represents an effective and potential genome editing tool in NIH/3T3 cell line. It was also shown that deletion of a gene fragment with high efficiency can occur in *Rag* genes by simultaneous cleavage at two targeted sites in one gene. Such results have been reported in other cases 8,30–33 but in this report significant deletion has been achieved in about 50% of cellular population. Taken together, these results showed that CRISPR/Cas9 system with highly active and correctly-designed sgRNAs generate mutations in desired genes and efficient deletions can be achieved using two exonic sgRNAs targeting one gene. Compared to frame shift mutations, genomic deletion can be useful for generation of specific gene knockouts.

The sequencing results demonstrated that the selected sgRNAs worked effectively with Cas9 on *Rag*1 and *Rag*2 genes in NIH/3T3 cell lines. *Rag*1/*Rag*2 gene targeting by CRISPR/Cas9 system makes deletions one-step in about 50% of cell lines. CRISPR/Cas9 system was applied with highly active sgRNAs for targeting the *Rag*1/*Rag*2 genes to make mutant cell lines. The CRISPR/Cas9 system is much easier than other systems like ZFN and TALEN to genome engineering. High efficiency editing by CRISPR-Cas system can be achieved in mouse cell line genomes at targeted locations with efficient and well-designed sgRNAs. Genome editing results indicated that CRISPR/Cas9 system with correctly-designed sgRNAs generate mutations in desired genes and significant deletions can be achieved in the large number of cells using two exonic sgRNAs targeting one gene.

## Conclusion

It was shown that CRISPR/Cas9 system can be highly efficient when gRNAs are designed rationally and provides a powerful approach for genetic engineering of cells and model animals.
